# Supplementary material for: SH3KBP1 promotes skeletal myofiber formation and functionality through ER/SR architecture integrity
Source: EMBO Rep. 2025 Mar 10;26(8):2166–91. doi: 10.1038/s44319-025-00413-9 (PMC12019163; doi:10.1038/s44319-025-00413-9)
Supplement: Supplementary file 7 — Expanded View Figures [file 44319_2025_413_MOESM7_ESM.pdf]

Expanded View Figures

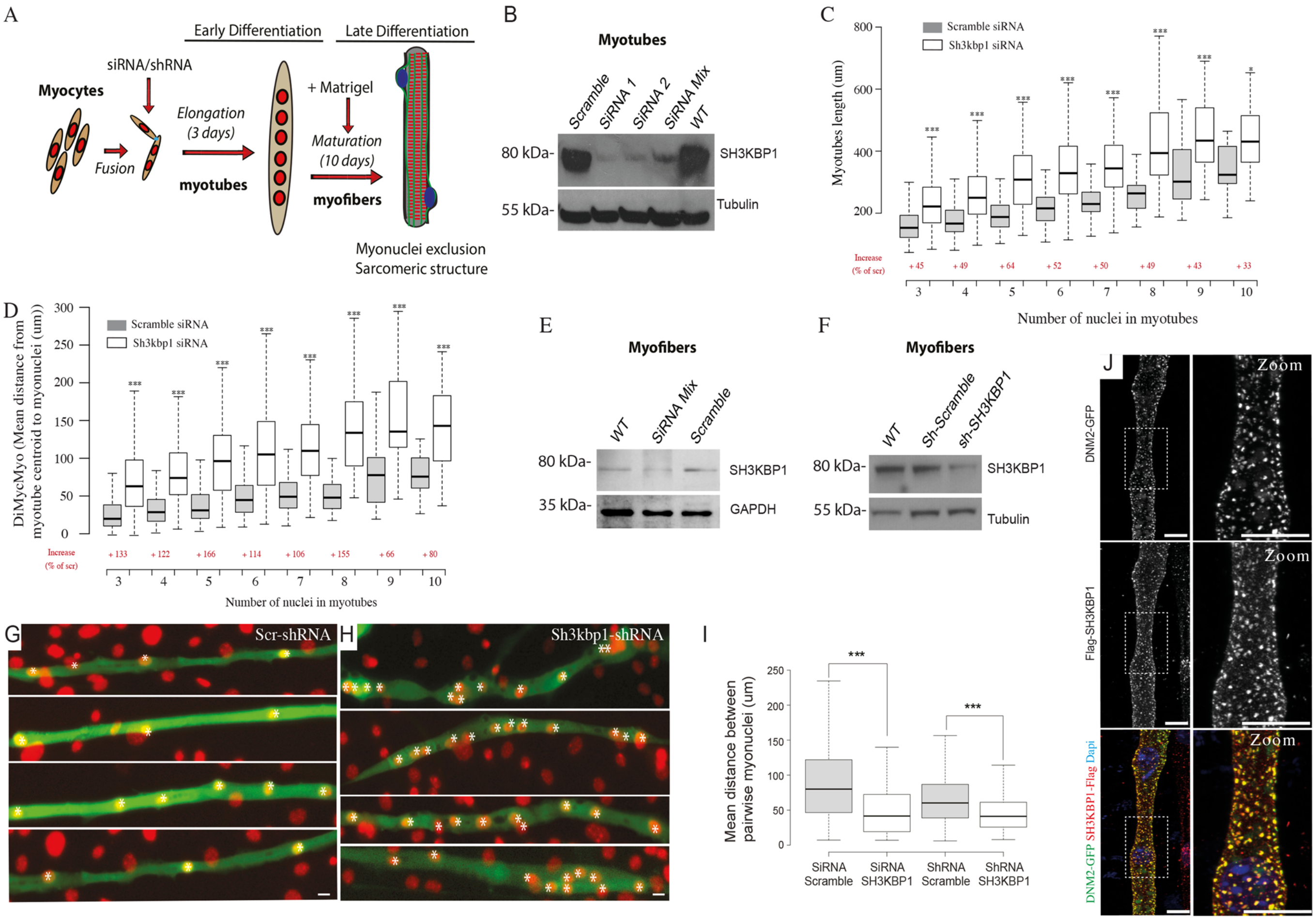

Figure EV1. SH3KBP1 affects myotubes elongation and myonuclei spreading.

(**A**) Sequential steps performed to obtain mature myofibers from primary myoblasts. siRNAs & shRNAs were transfected 24 h before myoblasts fusion. (**B**) Representative western blot analysis of SH3KBP1 protein levels in total protein extracts obtained after sh3kbp1 depletion using either 2 individual siRNA (1 & 2) or a pool of siRNA (Mix) after 3 days of differentiation of primary myoblasts; Tubulin used as loading control. (**C**) Distribution of myotubes length ranked by myonuclei content per myotubes quantified after 3 days of differentiation in cells treated with scramble or *Sh3kbp1* siRNAs. Comparison between Scramble siRNA and *sh3kbp1* siRNA for "3-nuclei" $P = 1{,}7E^{-26}$, "4-nuclei" $P = 9{,}9E^{-28}$, "5-nuclei" $P = 3{,}2E^{-22}$, "6-nuclei" $P = 6{,}35E^{-22}$, "7-nuclei" $P = 4{,}5E^{-15}$, "8-nuclei" $P = 2{,}1E^{-11}$, "9-nuclei" $P = 9{,}1E^{-7}$, "10-nuclei" $P = 0{,}03$. Statistical analysis performed using unpaired *t* tests where ***$P < 0.001$ and *$P < 0.05$. (**D**) Distribution of the mean distances between each myonuclei and myotube centroids (DiMycMyo) ranked by myonuclei content per myotubes were quantified after 3 days of differentiation in cells treated with scramble or *Sh3kbp1* siRNAs. Comparison between Scramble siRNA and *sh3kbp1* siRNA for "3-nuclei" $P = 1{,}9E^{-34}$, "4-nuclei" $P = 4{,}5E^{-38}$, "5-nuclei" $P = 3{,}7E^{-27}$, "6-nuclei" $P = 3{,}5E^{-23}$, "7-nuclei" $P = 1{,}2E^{-17}$, "8-nuclei" $P = 7{,}5E^{-15}$, "9-nuclei" $P = 1{,}3E^{-7}$, "10-nuclei" $P = 0{,}0006$. Statistical analysis performed using unpaired *t* tests where ***$P < 0.001$. (**C, D**) Data from five independent experiments were combined. Scramble siRNA cells ($n = 1010$ cells) and *Sh3kbp*1 siRNA cells ($n = 1093$ cells). Boxplot whiskers represent the maximum and minimum data values. Center lines show the medians; box limits indicate the 25th and 75th percentiles as determined by R software and represents the middle 50% of observed values. (**E, F**) Representative western blot analysis of SH3KBP1 protein levels in total protein extracts obtained after SH3KBP1 depletion using a pool of siRNA (Mix) or a shRNA targeting *sh3kbp1* after 10 days of differentiation of primary myoblasts (**E**, **F**, respectively); Tubulin or GAPDH used as loading control (**G**, **H**) Four representative images of 10 days differentiated myofibers transfected with either scramble (**G**) or *Sh3kbp1* shRNA tagged with GFP (**H**); shRNA (green) myonuclei (DAPI, red). Scale bar: 10 μm (asterisks are individual myonuclei). (**I**) Quantification of the mean distance between pairwise myonuclei in 10 days differentiated myofibers treated with scramble siRNA or shRNA, or with a pool of 2 individual siRNAs or of one individual shRNAs targeting *Sh3kbp1* ($n > 3$; biological replicates). Comparison between Scramble siRNA and *sh3kbp1* siRNA, $P = 3{,}1E^{-34}$; Comparison between Scramble shRNA and *sh3kbp1* shRNA, $P = 4{,}9E^{-34}$ Statistical analysis performed using unpaired *t* tests where ***$P < 0.001$. Boxplot whiskers represent the maximum and minimum data values. Center lines show the medians; box limits indicate the 25th and 75th percentiles as determined by R software and represents the middle 50% of observed values (**J**) Representative immunofluorescent staining of in vitro myofibers co-expressing GFP-DNM2 (green), SH3KBP1-Flag (red) and myonuclei (blue) in primary myotubes differentiated for 10 days. (Max intensity of Z stacks plans) Scale bars, 10 μm.

© The Author(s)

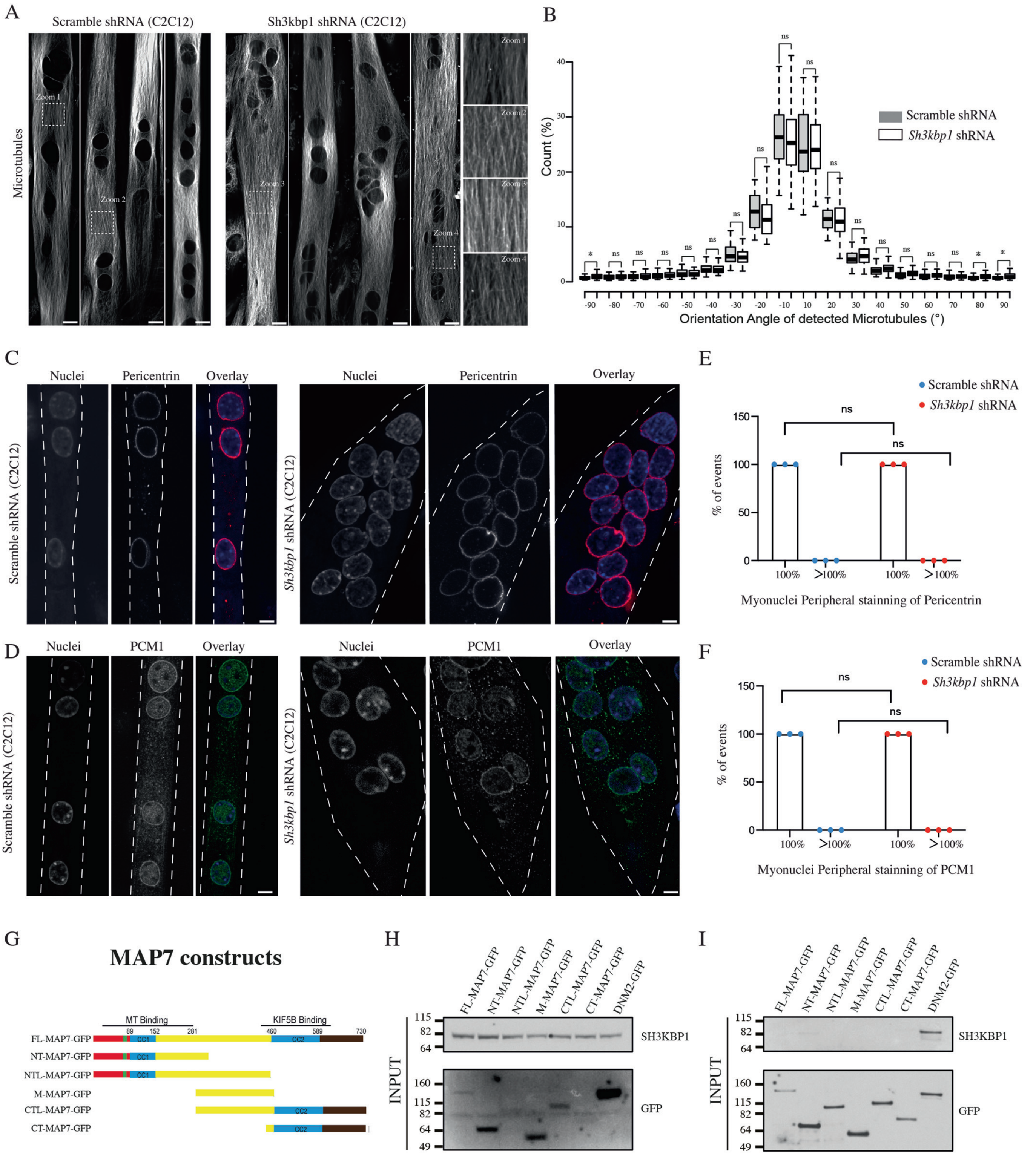
A
Scramble shRNA (C2C12)
Sh3kbp1 shRNA (C2C12)
Microtubules
Zoom 1
Zoom 2
Zoom 3
Zoom 4
B
Count (%)
Scramble shRNA
Sh3kbp1 shRNA
Orientation Angle of detected Microtubules (°)
C
Nuclei
Pericentrin
Overlay
Scramble shRNA (C2C12)
Sh3kbp1 shRNA (C2C12)
E
Scramble shRNA
Sh3kbp1 shRNA
% of events
Myonuclei Peripheral stainning of Pericentrin
D
Nuclei
PCM1
Overlay
F
Myonuclei Peripheral stainning of PCM1
G
MAP7 constructs
MT Binding
KIF5B Binding
FL-MAP7-GFP
NT-MAP7-GFP
NTL-MAP7-GFP
M-MAP7-GFP
CTL-MAP7-GFP
CT-MAP7-GFP
H
DNM2-GFP
INPUT
SH3KBP1
GFP
I

◀ **Figure EV2. SH3KBP1 is not affecting microtubule nucleation and organization in developing myotubes.**

(**A**) Representative immunofluorescence staining of 5 days C2C12 myotubes expressing either scramble or *Sh3kbp1* shRNA and stained with sirTubulin®. Scale bars, 10 µm. (**B**) Quantification of the microtubule bundle directionality (orientation angle normalized according to myotubes longitudinal axis) in myotubes using the "directionality plugin" of ImageJ®. Data are pooled from three independent repeats ($n = 41$ cells in scramble condition and $n = 61$ cells in *Sh3kbp1* shRNA condition). "−90°" category $P = 0.03$; "+80°" category $P = 0.02$" + 90°" category $P = 0.017$. Statistical analysis performed using unpaired t tests where $^*P < 0.05$. Boxplot whiskers represent the maximum and minimum data values. Center lines show the medians; box limits indicate the 25th and 75th percentiles as determined by R software and represents the middle 50% of observed values. (**C, D**) Representative images of immunofluorescent staining of Pericentrin (red), PCM1 (green) and nuclei (Blue) in 5 days differentiated C2C12 myotubes expressing either scramble or *Sh3kbp1* shRNA. Scale bars, 10 µm. (**E**, **F**) Quantification of the myonuclei peripheral staining of Pericentrin (**E**) or PCM1 (**F**) in 5 days differentiated C2C12 myotubes expressing either scramble or *Sh3kbp1* shRNA. Data are pooled from three independent repeats. Error bars represent SD (**G**) MAP7 constructs used in the experiment. (**H**) Representative western blot of crude extracts of C2C12 cells expressing various GFP-MAP7 constructs (FL: Full length, NT: N-terminal part of MAP7; NTL: N-terminal long part of MAP7; M: Middle part of MAP7, CT: C-terminal part of MAP7 and CTL: C-terminal long part of MAP7) and GFP-DNM2 and stained for endogenous SH3KBP1 (top) or with anti-GFP (bottom) antibodies. (**I**) Representative western blot after GFP immunoprecipitation (MAP7 and DNM2 constructs) using GFP-Trap in C2C12 cell extracts (**H**). The membrane was revealed with anti-GFP (bottom) and anti-SH3KBP1 (Top) antibodies n.>3.

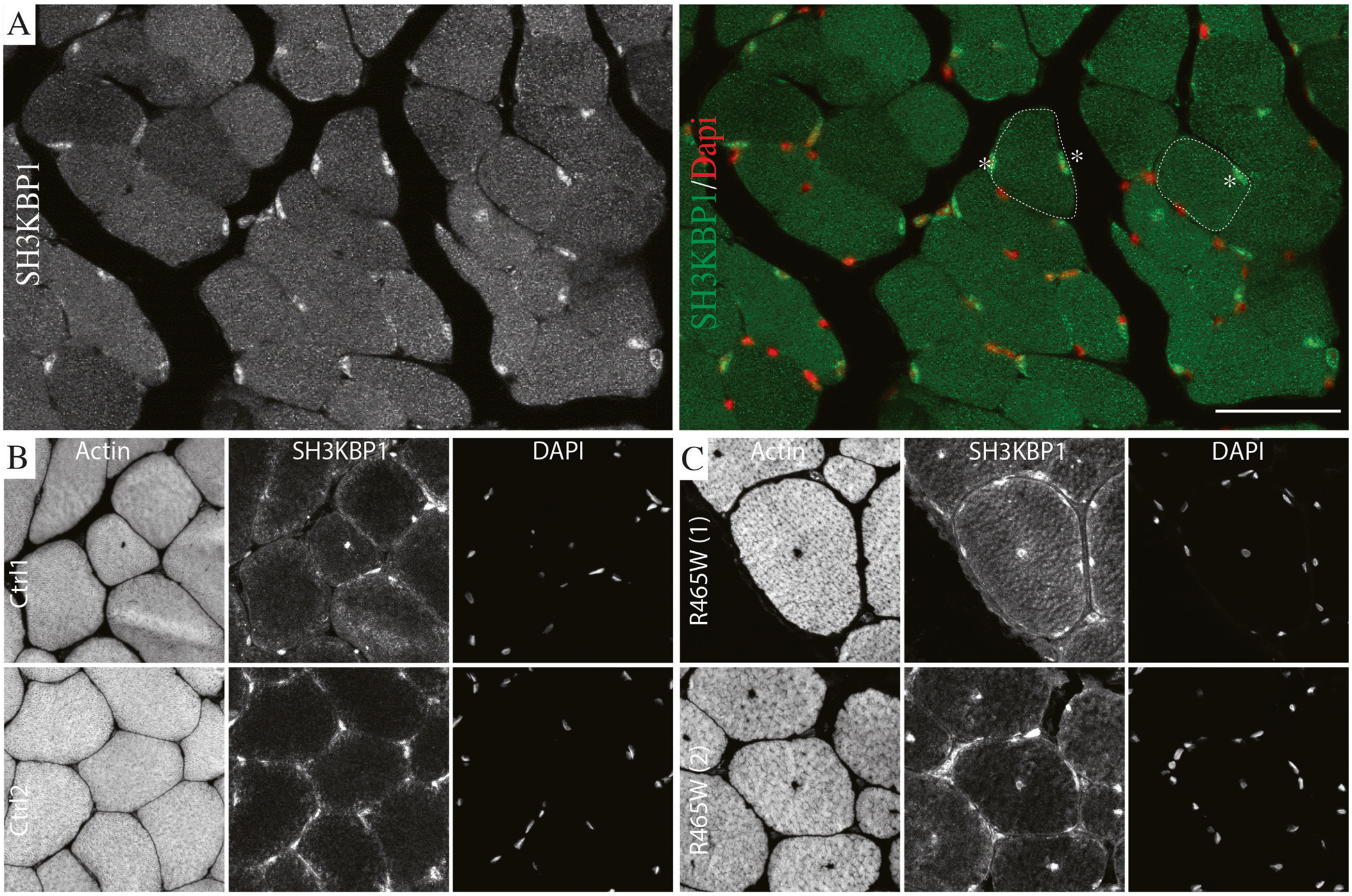

Figure EV3. SH3KBP1 is localized at myonuclei vicinity in mouse and human skeletal muscle fibers.

(**A**) Representative images of *Tibialis Anterior* muscle transversal cross-sections stained for SH3KBP1 (green) and myonuclei (Dapi, red). Asterisks show myonuclei inside myofibers. Scale bars, 150 μm. (**B**, **C**) Representative images of immunofluorescent staining of SH3KBP1, Actin and nuclei (Dapi) in transversal cross-sections of control human subject or of CNM patient harboring the p.R465W mutation. Scale bars, 150 μm.

 © The Author(s)

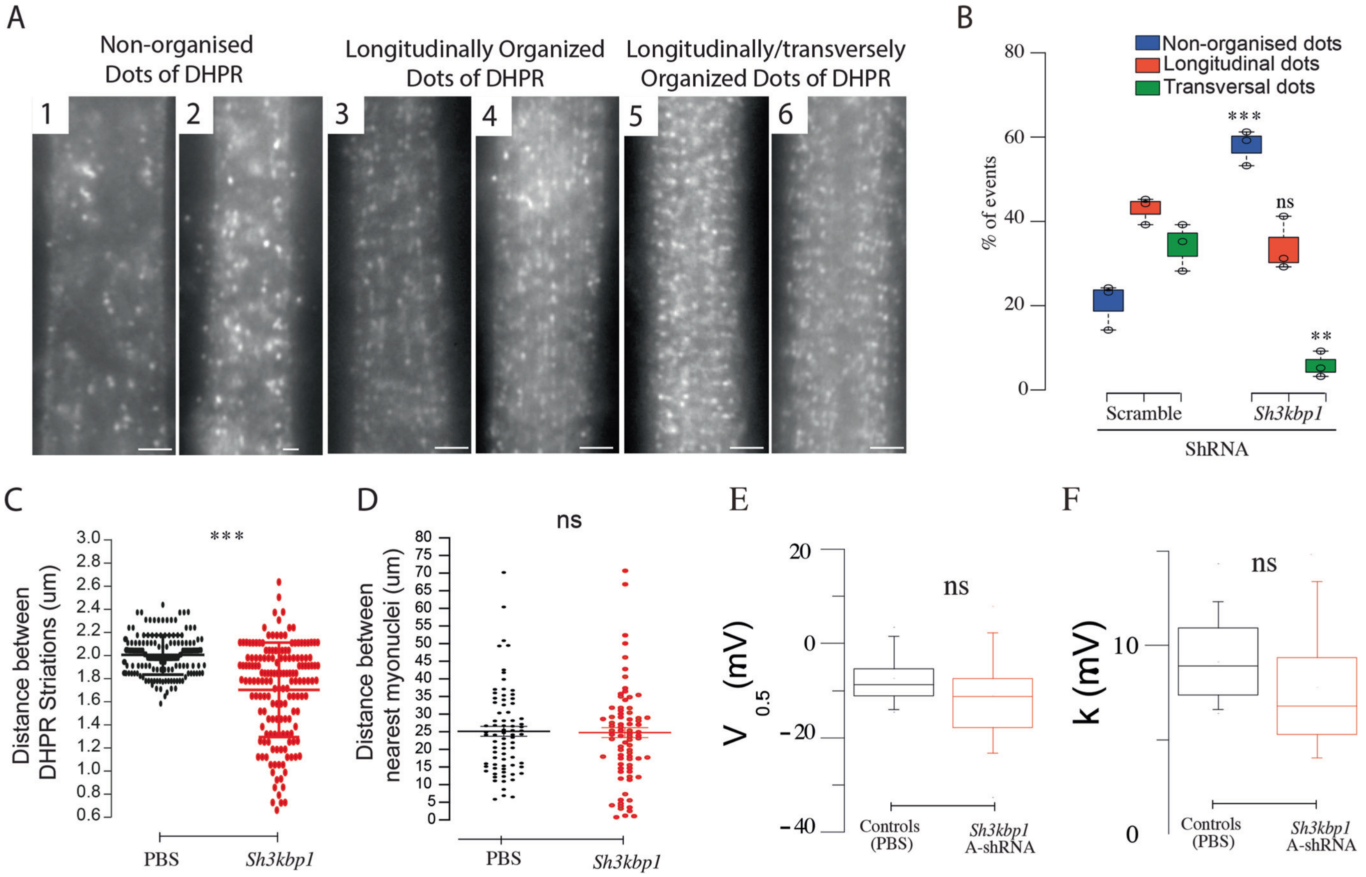

Figure EV4. SH3KBP1 contributes to T-tubule formation and Triads function

(**A**) Representative immunofluorescent images used for the classification of DHPR1-α staining patterns in 10 days cultured primary myotubes after scramble siRNA transfection. (1–2: non-organized DHPR1-α staining dots; 3-4: longitudinally organized DHPR1-α staining dots; 5–6: longitudinally and transversally organized DHPR1-α staining dots) Scale bars, 5 μm. (**B**) Quantification of DHPR1-α aspects in mature myofibers treated with either scramble shRNA or shRNA targeting *Sh3kbp1* mRNA. ($n = 3$; biological replicates) for the "non-organized DHPR dots" category $P = 0.0007$ and for the "transversal DHPR dots" category $P = 0.0015$. Statistical analysis performed using unpaired *t* tests where ***$P < 0.001$, **$P < 0.01$. Boxplot whiskers represent the maximum and minimum data values. Center lines show the medians; box limits indicate the 25th and 75th percentiles as determined by R software and represents the middle 50% of observed values. (**C**) Quantification of the mean distances between adjacent DHPR striations in *Tibialis Anterior* extracted muscle fibers from WT mice injected with either PBS or shRNA targeting *SH3KBP1* mRNA. ($n = 3$; biological replicates) with 166 measurements in scramble condition and 165 measurements in *Sh3kbp1* shRNA condition). $P = 4\ E^{-27}$. Error bars represent SD. Statistical analysis performed using unpaired *t* tests where ***$P < 0.001$. (**D**) Quantification of the distances between nearest myonuclei in *Tibialis Anterior* extracted muscle fibers from WT mice injected with either PBS or shRNA targeting *SH3KBP1* mRNA. (**E, F**) Half-activation (**E**) and steepness factor (**F**) for SR Ca^{2+} release in the two groups of fibers, as assessed from Boltzmann fits to data from each fiber. Boxplot whiskers represent the maximum and minimum data values. Center lines show the medians; box limits indicate the 25th and 75th percentiles as determined by R software and represents the middle 50% of observed values.

© The Author(s)

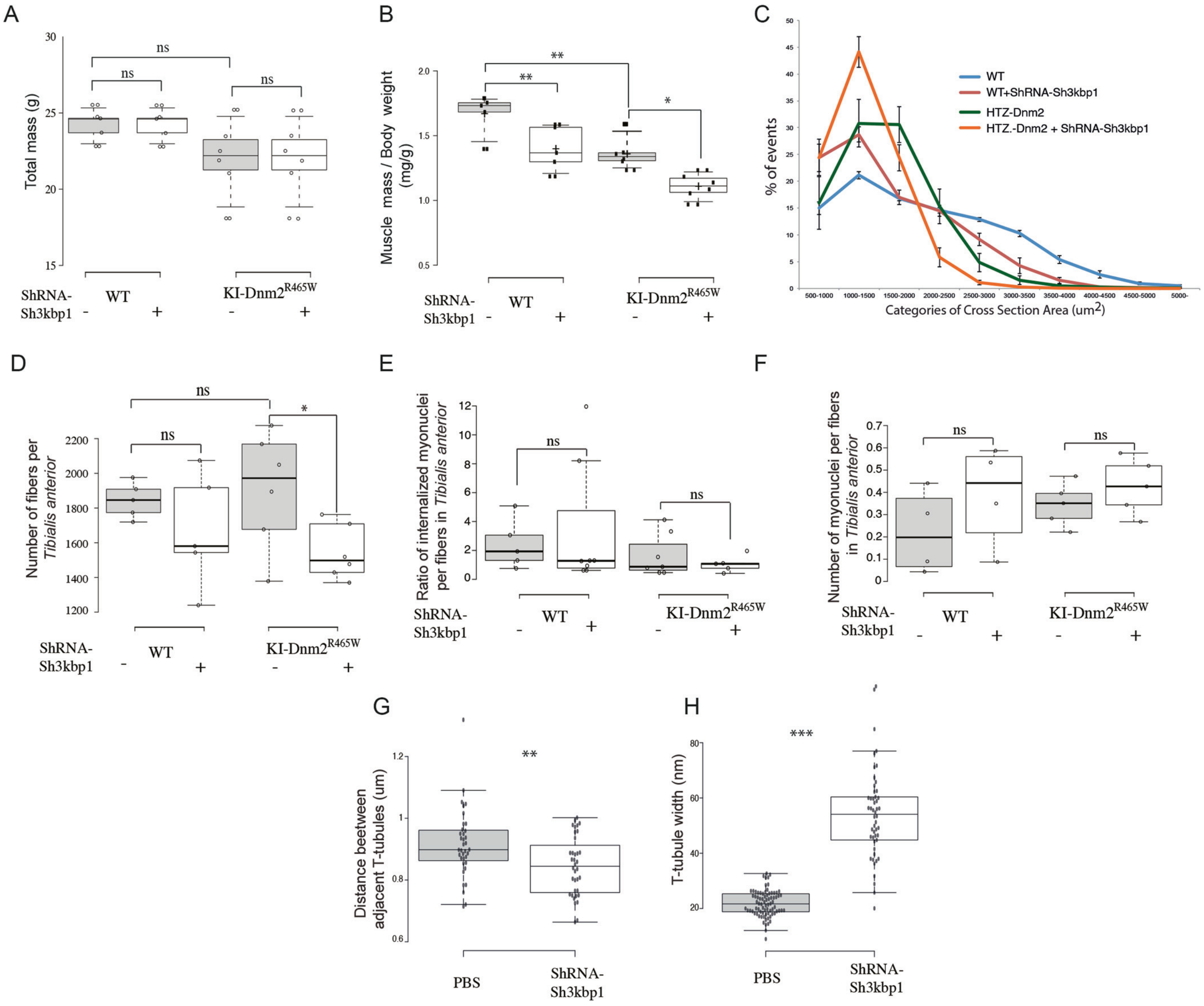

Figure EV5. ***Sh3kbp1* silencing affects myofibers parameters in WT or in KI-*Dnm2*$^{R465W/+}$ mice model.**

(**A**) Quantification of total body mass from WT or KI-*Dnm2*$^{R465W/+}$ mice at the age of 4 months, injected with either PBS or AAV cognate vector expressing shRNA targeting SH3KBP1 mRNA (AAV-SH3KBP1) for 3 months. Number of mice per group: $n = 8$. (**B**), Quantification of muscle mass normalized to respective mice body weight from WT or KI-*Dnm2*$^{R465W/+}$ mice injected with either PBS or AAV-SH3KBP1 for 3 months. ($n > 7$; biological replicates) Comparison between WT and WT-ShRNA-*sh3kbp1* $P = 0.003$, WT and KI-DNM2^{R465W} $P = 0.004$, KI-DNM2^{R465W} and KI-DNM2^{R465W}-ShRNA-*sh3kbp1* $P = 0.01$. Statistical analysis performed using unpaired *t* tests where $^{**}P < 0.01$ and $^{*}P < 0.05$. (**C**) Distribution of cross-sectional myofibers areas in *Tibialis Anterior* muscles from WT or KI-*Dnm2*$^{R465W/+}$ mice injected with either PBS or AAV-SH3KBP1 for 3 months. ($n = 4$; biological replicates) Error bars represent SD. (**D**) Quantification of the number of fibers per *Tibialis Anterior* muscles from WT or KI-*Dnm2*$^{R465W/+}$ mice injected with either PBS or AAV-SH3KBP1 for 3 months. ($n > 5$; biological replicates) Statistical analysis performed using unpaired *t* tests where $^{*}P < 0.05$. (**E**) Quantification of internalized myonuclei in *Tibialis Anterior* muscles myofibers from WT or KI-*Dnm2*$^{R465W/+}$ mice injected with either PBS or AAV-SH3KBP1 for 3 months. ($n > 5$; biological replicates). (**F**) Quantification of the number of myonuclei per fibers in *Tibialis Anterior* muscles myofibers from WT or KI-*Dnm2*$^{R465W/+}$ mice injected with either PBS or AAV-SH3KBP1 for 3 months. ($n > 5$; biological replicates). (**G**, **H**) Quantification of the distances between adjacent T-tubules structures in *Tibialis Anterior* muscles extracted from WT or KI-*Dnm2*$^{R465W/+}$ mice injected with either PBS or AAV-SH3KBP1 for 3 months. $P = 0.004$. Two mice were combined for each condition ($n = 36$ measurements per condition), Statistical analysis performed using unpaired *t* tests where $^{**}P < 0.01$. (**G**) Quantification of individual T-tubule width in *Tibialis Anterior* muscles extracted from WT or KI-*Dnm2*$^{R465W/+}$ mice injected with either PBS or AAV-SH3KBP1 for 3 months. $P = 1{,}6$ E^{-34}. Two mice were combined for each condition ($n = 79$ measurements in Control condition and $n = 50$ in AAV-SH3KBP1 condition) Statistical analysis performed using unpaired *t* tests where $^{***}P < 0.001$. (**A**, **B**, **D–H**) Boxplot whiskers represent the maximum and minimum data values. Center lines show the medians; box limits indicate the 25th and 75th percentiles as determined by R software and represents the middle 50% of observed values.

 © The Author(s)
